# Epstein-Barr Virus miRNAs in Multiple Sclerosis: Unveiling their role in Immune Regulation and Potential for Diagnostic and Therapeutic Innovation

**DOI:** 10.12669/pjms.41.10.12579

**Published:** 2025-10

**Authors:** Eda Balkan, Murat Kızılkaya, Nuray Bilge, Merve Aykaç, Filiz Demirdoğan

**Affiliations:** 1 Eda Balkan Department of Medical Biology, Atatürk University, Erzurum, Türkiye; 2 Murat Kızılkaya Department of Medical Biology, Ağrı İbrahim Çeçen University, Ağrı, Türkiye; 3 Nuray Bilge Department of Neurology, Atatürk University, Erzurum, Türkiye; 4 Merve Aykaç Department of Medical Biology, Atatürk University, Erzurum, Türkiye; 5 Filiz Demirdoğan Department of Neurology, Atatürk University, Erzurum, Türkiye

**Keywords:** Epstein-Barr Virus, multiple sclerosis, miRNA

## Abstract

**Objective:**

This study investigates the role of Epstein-Barr virus (EBV)-derived microRNAs (miRNAs) in the pathogenesis of multiple sclerosis (MS), aiming to elucidate their impact on immune-related molecular mechanisms.

**Methodology:**

This study was conducted at Ataturk University (Departments of Neurology and Medical Biology), between July 2022 and March 2025, the study included 40 MS patients and 40 healthy controls. Peripheral blood RNA samples were analyzed using Real-Time PCR to measure EBV miRNA and host mRNA expression. Data were evaluated using SPSS v23.

**Results:**

EBV-derived miRNA levels were significantly elevated in MS patients. Specifically, BART17-5p, BART10-5p, BHRF1-3 and BART5-3p levels were markedly increased (p<0.001). BART8-5p showed a negative correlation with CREB1 (r=-0.419; p=0.021), suggesting suppression of host signaling pathways. Immune markers such as CXCL3, MALT1, IFNB1, IL6, IL2 and FOXP3 also differed significantly between groups (p=0.005). Moderate correlations were noted between EBV-miRNAs and immune genes (e.g., IFNB1 and BART10-5p: r=0.433; p=0.017). Weak negative correlations were found between BART8-5p and multiple immune-related genes (e.g., IL1B, STAT3, CD4) and between BART19 and IL2 (r=-0.373; p=0.043). Logistic regression revealed that increased BART miRNA levels were associated with elevated MS risk.

**Conclusion:**

EBV-derived miRNAs are linked to immune activation and may contribute to MS pathogenesis. These miRNAs may serve as potential biomarkers and therapeutic targets in MS management.

## INTRODUCTION

Multiple sclerosis (MS) is a chronic autoimmune disease of the central nervous system, characterized by inflammation, demyelination and neurodegeneration. It primarily affects young adults, especially females and is classified into clinical subtypes such as RRMS, SPMS, PPMS and PRMS.^1^ Both genetic and environmental factors contribute to MS pathogenesis. Among environmental triggers, Epstein-Barr virus (EBV) infection has been identified as a strong risk factor EBV seropositivity has been reported in approximately 95–99% of MS patients, supporting a robust epidemiological association between EBV infection and MS.^1^ EBV is a herpesvirus that persists in a latent state and modulates host immunity. It encodes over 40 microRNAs (miRNAs) capable of interfering with host gene expression, and has also been associated with various malignancies, including oral squamous cell carcinoma.^2^-^4^

**Fig.1:**
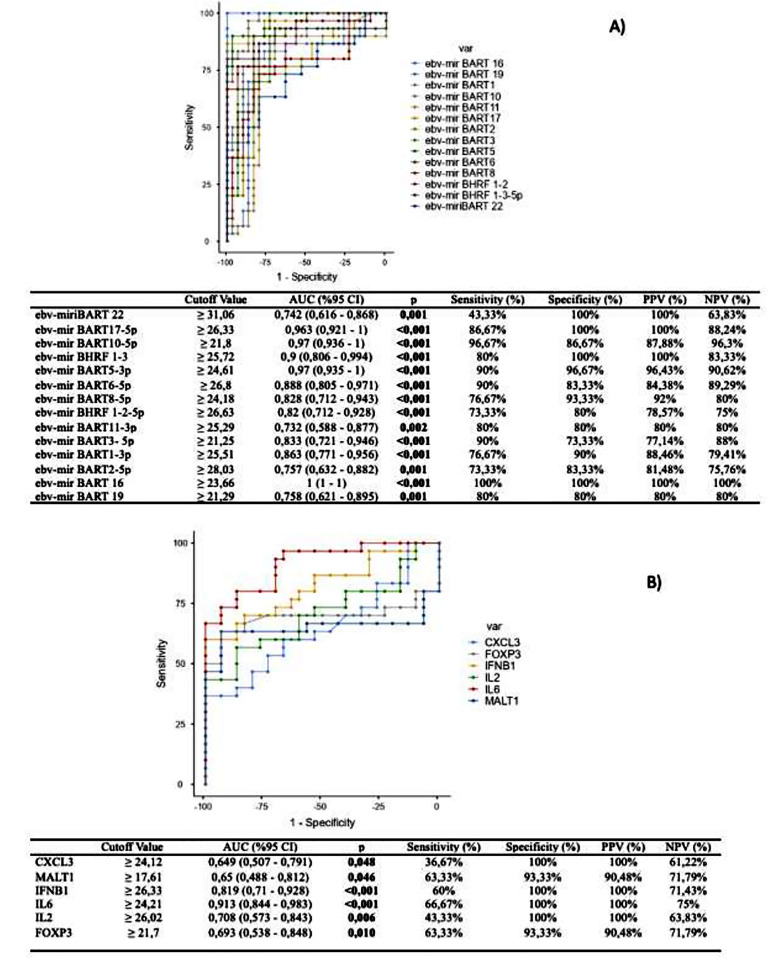
A) ROC Analysis Results: AUC Value of EBV miRNA. B) ROC Analysis Results: AUC Value of EBV-positive MS patient miRNA.

MiRNAs are small non-coding RNAs that regulate gene expression post-transcriptionally. Dysregulation of miRNAs is implicated in autoimmune diseases, including MS. EBV miRNAs, in particular, may contribute to immune evasion, inhibition of apoptosis and B-cell transformation.^5^-^7^

The BART and BHRF1 regions of the EBV genome encode miRNAs that are expressed in different latency forms. These miRNAs may affect key pathways related to MS by targeting immune-related genes.^8^-^12^

In this study, we examined how EBV-encoded miRNAs interact with host mRNAs involved in MS. Our aim was to clarify their potential role in MS development and to identify novel molecular targets for diagnosis and therapy.^13^,^14^

## METHODOLOGY

This study was conducted at Atatürk University in collaboration with the Departments of Medical Biology and Neurology, between July 2022 and March 2025 It enrolled 40 treatment-naive adults with relapsing-remitting MS (RRMS), diagnosed per the 2010 revised McDonald criteria and 30 age- and sex-matched EBV-seropositive healthy controls without neurological or systemic disorders. Peripheral blood samples were collected before treatment to evaluate EBV serostatus, EBNA profile, immune markers and molecular signatures. Clinical data-including age, sex, disease duration and EDSS scores-were extracted from medical records and questionnaires. Inclusion required full EBV data and absence of prior immunomodulatory therapy.

### Ethical Approval:

It was granted by the Atatürk University Faculty of Medicine Ethics Committee (Approval No: B56.6 ATA-0.01.00/6-56:2022) June 30^th^ 2022 and written informed consent was obtained from all participants.

Peripheral blood samples were collected before treatment. Total RNA was isolated using the QIAGEN RNeasy Plus Mini Kit with DNase treatment. RNA quality was assessed via spectrophotometry and gel electrophoresis. For expression profiling, miRNA cDNA was synthesized using the iScript Universal Kit and mRNA cDNA using the QIAGEN QuantiTect RT Kit. Quantitative real-time PCR was performed using SYBR Green chemistry on a Rotor-Gene system, with 40 amplification cycles and melting curve analysis. Expression levels were calculated by the ΔCT method and normalized to GAPDH. All reactions were done in duplicates. Statistical analyses were conducted using SPSS v23. Data distribution was tested with Shapiro-Wilk; group comparisons used t-test or Mann-Whitney U. Correlations were assessed via Pearson or Spearman tests. Logistic regression and ROC analysis evaluated diagnostic value. Significance was set at p < 0.05.

## RESULTS

The mean age of MS patients was 32.1 ± 8.1 years and the mean disease duration was 5.1 ± 4.2 years. The mean EDSS score was 1.95 ± 1.27. A significant increase was observed in the expression levels of EBV-derived miRNAs in MS patients, particularly EBV-miRBART17-5p, EBV-miRBART10-5p, EBV -miRBHRF1-3 and EBV -miRBART5-3p (p < 0.001). Expression levels of immune-related genes such as CXCL3, MALT1, IFNB1, IL6, IL2 and FOXP3 also differed significantly between groups (p < 0.05).

Correlation analysis showed a significant positive correlation between IFNB1 and EBV-miR BART10-5p (p=0.017) and significant negative correlations between EBV-miR BART8-5p and CREB1, CXCL3, CD4, E2F3, STAT3, MICB and IL1B (p<0.05). EBV-miR BART19 was negatively correlated with IL2 , p = 0.043).

In MS patients, a significant positive correlation was found between IFNB1 and EBV-miR BART10-5p (r = 0.433, p = 0.017). EBV-miR BART8-5p showed negative correlations with CREB1 (r = -0.419, p = 0.021), CXCL3 (r = -0.429, p = 0.018) and CD4 (r = -0.426, p = 0.019). Additional weak negative correlations were observed between EBV-miR BART8-5p and E2F3, STAT3, MICB and IL1B (p < 0.05). EBV-miR BART19 was negatively correlated with IL2 (r = -0.373, p = 0.043). No significant correlations were observed for other parameters.

EBV-miR EBV-miR EBV-miR BART22, EBV-miR BART17-5p, EBV-miR BART10-5p, EBV-miR BHRF1-3 and EBV-miR BART5-3p) significantly elevated the risk of developing multiple sclerosis (p < 0.05). For instance, a one-unit increase in EBV-miR BART17-5p and EBV-miR BART10-5p expression raised disease risk by more than twofold. Similarly, higher expression of host immune genes including IL6, IFNB1, IL1B, IL2, FOXP3 and CXCL3 was also associated with increased MS risk. IL6 emerged as a particularly strong predictor, with a one-unit increase nearly tripling the disease risk (OR = 2.885; p < 0.001

## DISCUSSIONS

This study supports the growing body of evidence implicating Epstein-Barr virus (EBV) in the immunopathogenesis of multiple sclerosis (MS) through the expression of viral microRNAs (miRNAs). Consistent with previous studies, we observed significantly elevated levels of EBV-derived miRNAs-especially *BART17-5p*, *BART10-5p*, *BHRF1-3* and *BART5-3p*-in relapsing-remitting MS (RRMS) patients compared to healthy controls (all p < 0.001), suggesting their potential as disease biomarkers.^15^-^17^

Our data align with reports by Wang et al., who demonstrated that BART and BHRF1 miRNAs facilitate viral immune evasion by downregulating host antiviral responses.^18^ Notably, we observed increased expression of *IL6*, *IFNB1* and *FOXP3* in patients, which may reflect EBV-induced modulation of both inflammatory and regulatory immune pathways. The positive correlation between *EBV-miR BART10-5p* and *IFNB1* and the negative correlation between *BART8-5p* and *CREB1* are particularly relevant, as *CREB1* is known to modulate neuroinflammation.

**Table-I:** Comparison of EBV miRNA and Host mRNA Expression Levels Between Multiple Sclerosis Patients and Healthy Controls.

	Groups		p
	Control	Patients	
***MS and Control***			
ebv-miriBART 22	23,5 (8,42 - 30,91)	29,5 (12,84 - 36,92)	0,001*
ebv-mir BART17-5p	20,13 (16,83 - 25,78)	29,74 (20,12 - 34,61)	<0,001*
ebv-mir BART10-5p	15,9 (11,57 - 25,4)	27,96 (21,29 - 33,95)	<0,001*
ebv-mir BHRF 1-3	17,72 (13,87 - 25,15)	30,07 (11,59 - 36,9)	<0,001*
ebv-mir BART5-3p	18,82 ± 3,49	28,92 ± 4,39	<0,001**
ebv-mir BART6-5p	17,83 (12,45 - 32,24)	30,64 (17,97 - 36,33)	<0,001*
ebv-mir BART8-5p	18,34 (10,22 - 25,15)	28,31 (13,37 - 37,56)	<0,001*
ebv-mir BHRF 1-2-5p	18,61 (12,9 - 35,19)	32,76 (14,7 - 36,41)	<0,001*
ebv-mir BART11-3p	19,62 (14,91 - 37,39)	30,95 (11,97 - 37,67)	0,002*
ebv-mir BART3- 5p	16,83 (11,36 - 33,77)	29,29 (10,88 - 37,84)	<0,001*
ebv-mir BART1-3p	18,76 (11,56 - 30,71)	30,07 (15,79 - 35,73)	<0,001*
ebv-mir BART2-5p	23,63 (12,95 - 34,22)	29,83 (15,88 - 35,06)	0,001*
ebv-mir BART 16	19,09 ± 1,4	29 ± 2,87	<0,001**
ebv-mir BART 19	17,84 (12,34 - 34,36)	27,93 (11,86 - 35,65)	0,001*
***EBV + MS Patients***			
CREB1	16,57 (14,89 - 17,81)	16,97 (14,09 - 26,23)	0,104*
E2F3	17,57 (16,62 - 20,42)	17,55 (11,76 - 27,68)	0,487*
CXCL3	21,53 (18,2 - 23,85)	22,32 (18,51 - 27,41)	0,047*
MALT1	16,94 (15,6 - 17,78)	17,7 (14,47 - 28,26)	0,046*
IFNB1	24,01 (13,49 - 25,79)	27,28 (21,39 - 30,13)	<0,001*
IL6	21,04 ± 1,53	24,76 ± 2,08	<0,001**
STAT3	15,26 (13,49 - 16,75)	15,38 (9,28 - 25,74)	0,871*
MICB	18,42 (16,6 - 19,47)	18,24 (15,23 - 25,36)	0,935*
IL1B	16,08 (12,6 - 17,6)	16,25 (12,4 - 27,97)	0,767*
IL2	23,79 (17,2 - 25,96)	25,34 (20,76 - 29,8)	0,006*
FOXP3	20,55 ± 0,65	22,71 ± 3,1	0,001**
CD4	16,66 (15,11 - 20,67)	16,7 (12,75 - 24,14)	0,762*

**Table-II:** Correlation Analysis Between Viral miRNAs and Host mRNA Expression Levels in EBV-Positive MS Patients and Healthy Control.

		CREB1	E2F3	CXCL3	MALT1	IFNB1	IL6	STAT3	MICB	IL1B	IL2	FOXP3	CD4
***Patients***													
ebv-mir BART 19	r	-0,064	0,073	0,020	-0,211	0,382	0,274	-0,100	0,173	0,006	0,181	0,100	0,212
	p	0,737	0,703	0,914	0,264	0,037	0,143	0,600	0,360	0,977	0,339	0,601	0,260
ebv-miriBART 22	r	0,032	0,057	0,021	0,019	0,122	0,055	0,044	0,039	0,044	-0,126	-0,060	0,097
	p	0,865	0,765	0,911	0,922	0,520	0,773	0,817	0,839	0,818	0,507	0,753	0,610
ebv-mir BART17-5p	r	0,218	0,256	0,360*	0,229	0,042*	0,123*	0,247	0,318	0,298	0,128*	0,319*	0,253
	p	0,246	0,172	0,051	0,223	0,825	0,519	0,188	0,087	0,109	0,499	0,086	0,178
ebv-mir BART10-5p	r	0,170	0,108	0,124	0,118	0,433	0,238	0,110	0,125	0,107	0,067	0,068	0,055
	p	0,369	0,572	0,514	0,535	0,017	0,205	0,564	0,509	0,574	0,727	0,720	0,773
ebv-mir BHRF 1-3	r	0,000	-0,041	0,006	0,018	0,155	-0,021	-0,055	-0,019	-0,014	0,076	0,025	-0,054
	p	1,000	0,828	0,973	0,925	0,413	0,912	0,772	0,922	0,940	0,690	0,897	0,778
ebv-mir BART5-3p	r	-0,152	-0,074	-0,171*	-0,109	0,144*	-0,045*	-0,165	-0,123	-0,188	0,007*	-0,174*	-0,128
	p	0,422	0,699	0,366	0,565	0,446	0,814	0,385	0,516	0,320	0,972	0,359	0,500
ebv-mir BART6-5p	r	0,090	0,204	0,054	0,109	-0,026	0,024	0,148	0,224	0,151	-0,248	0,097	0,151
	p	0,637	0,280	0,778	0,568	0,891	0,901	0,434	0,235	0,425	0,187	0,612	0,424
ebv-mir BART8-5p	r	-0,419	-0,380	-0,429	-0,343	-0,121	-0,100	-0,369	-0,369	-0,395	-0,069	-0,334	-0,426
	p	0,021	0,038	0,018	0,063	0,526	0,599	0,045	0,045	0,031	0,717	0,072	0,019
ebv-mir BHRF 1-2-5p	r	0,093	0,061	0,058	0,083	0,120	0,048	0,067	-0,061	0,014	-0,033	-0,004	0,031
	p	0,623	0,749	0,760	0,664	0,529	0,802	0,724	0,750	0,942	0,863	0,982	0,872
ebv-mir BART11-3p	r	-0,048	-0,099	-0,090	-0,106	0,061	0,047	-0,075	-0,139	-0,151	-0,133	-0,090	-0,125
	p	0,802	0,601	0,636	0,578	0,748	0,806	0,692	0,464	0,427	0,484	0,637	0,511
ebv-mir BART3- 5p	r	-0,044	-0,135	-0,015	-0,094	-0,189	-0,174	-0,056	-0,174	-0,100	-0,021	-0,094	-0,092
	p	0,817	0,477	0,937	0,622	0,317	0,358	0,767	0,358	0,599	0,911	0,620	0,628
ebv-mir BART1-3p	r	0,208	0,159	0,285	0,217	-0,078	-0,090	0,179	0,161	0,254	0,125	0,161	0,208
	p	0,269	0,401	0,127	0,250	0,681	0,637	0,345	0,395	0,176	0,511	0,396	0,269
ebv-mir BART2-5p	r	0,076	0,063	0,129	0,060	0,289	0,147	0,104	0,059	0,072	0,037	0,020	0,040
	p	0,691	0,740	0,496	0,751	0,122	0,439	0,585	0,756	0,704	0,847	0,917	0,832
ebv-mir BART 16	r	0,018	-0,044	0,106*	-0,042	0,184*	-0,085*	-0,065	0,040	-0,038	-0,059*	-0,012*	-0,054
	p	0,924	0,818	0,579	0,824	0,331	0,653	0,734	0,833	0,843	0,757	0,951	0,777
ebv-mir BART 19	r	-0,268	-0,270	-0,223	-0,280	-0,071	-0,161	-0,238	-0,300	-0,211	-0,373	-0,311	-0,291
	p	0,153	0,149	0,236	0,135	0,711	0,396	0,205	0,108	0,264	0,043	0,095	0,119

**Table-III:** Examination of Associations Between EBV-miRNAs and Host mRNAs in Multiple Sclerosis Patients and Controls Using Binary Logistic Regression.

	Groups		Univariate	
	Control	Patients	OR (%95 CI)	p
ebv-miriBART 22	22,48 ± 6,45	28,06 ± 6,96	1,133 (1,039 - 1,236)	0,005
ebv-mir BART17-5p	20,98 ± 3,11	29,62 ± 3,49	2,051 (1,376 - 3,057)	<0,001
ebv-mir BART10-5p	17,19 ± 3,89	28,24 ± 4,24	2,165 (1,328 - 3,529)	0,002
ebv-mir BHRF 1-3	19,02 ± 4,22	29,36 ± 6,36	1,356 (1,173 - 1,567)	<0,001
ebv-mir BART5-3p	18,82 ± 3,49	28,92 ± 4,39	2,159 (1,408 - 3,311)	<0,001
ebv-mir BART6-5p	20,3 ± 6,13	30,16 ± 4,22	1,346 (1,171 - 1,547)	<0,001
ebv-mir BART8-5p	18,28 ± 4,89	27,67 ± 7,75	1,236 (1,109 - 1,378)	<0,001
ebv-mir BHRF 1-2-5p	21,55 ± 6,59	29,64 ± 6,12	1,192 (1,089 - 1,304)	<0,001
ebv-mir BART11-3p	22,96 ± 7,23	29,23 ± 7,12	1,124 (1,041 - 1,213)	0,003
ebv-mir BART3- 5p	17,98 ± 5,77	27,23 ± 6,92	1,228 (1,108 - 1,36)	<0,001
ebv-mir BART1-3p	19,17 ± 5,47	28,01 ± 5,39	1,301 (1,147 - 1,474)	<0,001
ebv-mir BART2-5p	23,11 ± 6,05	28,58 ± 5,02	1,19 (1,069 - 1,324)	0,001
ebv-mir BART 16	19,09 ± 1,4	29 ± 2,87	---	---
ebv-mir BART 19	19,52 ± 6,13	26,17 ± 6,93	1,157 (1,061 - 1,262)	0,001
CREB1	16,56 ± 0,78	18,63 ± 3,89	1,367 (1,053 - 1,774)	0,019
E2F3	17,93 ± 1,05	18,58 ± 4,17	1,075 (0,905 - 1,278)	0,410
CXCL3	21,42 ± 1,58	22,93 ± 2,42	1,461 (1,089 - 1,958)	0,011
MALT1	16,9 ± 0,55	19,14 ± 3,96	1,438 (1,073 - 1,927)	0,015
IFNB1	23 ± 3,52	26,58 ± 2,38	1,851 (1,315 - 2,605)	<0,001
IL6	21,04 ± 1,53	24,76 ± 2,08	2,885 (1,72 - 4,84)	<0,001
STAT3	15,26 ± 0,91	17,04 ± 4,53	1,202 (0,998 - 1,447)	0,053
MICB	18,22 ± 0,77	19,42 ± 3,23	1,263 (0,986 - 1,617)	0,064
IL1B	16,1 ± 0,99	18,23 ± 4,74	1,225 (1,016 - 1,477)	0,033
IL2	23,29 ± 2,36	25,46 ± 2,68	1,45 (1,118 - 1,881)	0,005
FOXP3	20,55 ± 0,65	22,71 ± 3,1	1,663 (1,174 - 2,356)	0,004
CD4	17,21 ± 1,5	17,88 ± 3,53	1,098 (0,905 - 1,332)	0,344

Similar to Afrasiabi et al.^13^, who identified EBV miRNAs targeting *IL7R* and *IL2RA*, we found altered expression of *MALT1*, *CXCL3* and *FOXP3*, suggesting EBV miRNA-mediated regulation of immune genes relevant to MS. Moreover, our results support findings from Rang et al., who showed that EBV miRNAs modulate NF-κB and PD-1/PD-L1 signaling pathways, as we observed altered expression of *MALT1* and *IFNB1*.^19^ ROC analyses in our study and in the work of Y.F. Wang et al.^20^ similarly highlight *BHRF1-2-5p* and *BHRF1-3* as promising diagnostic markers.

### Limitations:

However, the study is limited by its small sample size, single-center design, and the absence of an EBV-negative control group, which restricts interpretation of virus-specific effects. Furthermore, our targeted approach did not include whole-transcriptome or advanced network analyses. Therefore, to establish causal relationships and validate these results, larger, multi-center, and longitudinal studies incorporating comprehensive profiling and EBV-negative controls are warranted.

## CONCLUSION

In this study, the EBV-miRNAs analyzed may contribute to MS pathogenesis through mechanisms such as modulation of immune responses, inhibition of apoptosis, and activation of B cells. Our findings suggest that these miRNAs could serve as potential diagnostic biomarkers and therapeutic targets for MS.
